# Medical education: simulation and virtual reality

**DOI:** 10.1590/S1516-31802011000600001

**Published:** 2011-12-01

**Affiliations:** I MD. Thoracic Surgeon, Instituto Dante Pazzanese de Cardiologia, and Postgraduate Student in the Discipline of Thoracic and Cardiovascular Surgery, Faculdade de Medicina da Universidade de São Paulo (FMUSP), São Paulo, Brazil.; II MD, PhD. Associate Professor, Discipline of Thoracic Surgery, Instituto do Coração (InCOR), Hospital das Clínicas (HC), Faculdade de Medicina da Universidade de São Paulo (FMUSP), São Paulo, Brazil.

Medical training in general, and surgical training especially, has always been based on learning the theory, followed by clinical experience gained through students’ direct contact with patients. Technological evolution within education is progressively but definitively giving rise to a new stage in this process: simulation.

Simulation as a form of learning gained strength in the 1930s through the popularization of the first flight simulator, which was known as the “Link Trainer”. The idea of teaching in simulated situations quickly drew the attention of people involved in teaching medicine, but because of the technical difficulties and cost, no great advances were viable at that time. The first simulator within medicine that achieved widespread acceptance was developed at the beginning of the 1960s by Åsmund Laerdal, a manufacturer of plastic toys. His simulator consisted of a dummy that was designed to provide training for mouth-to-mouth resuscitation; it became known as “Resusci Anne”. It was so successful that the product continued to be refined over the years, to reach the present day with a variety of improvements that enable effective training for dealing with cardiorespiratory arrest.^1^

The history of computing and virtual reality in medical training began with a theoretical proposal for a man-machine graphical interface called the “Sketchpad”, made by Ivan Sutherland in the 1960s. Nevertheless, advances in this field only started to accelerate in the 1980s, with the proliferation of computers and peripherals. In 1989, two NASA (National Aeronautics and Space Administration) employees presented the device that became known as the first computer-based surgical simulator. This device could be used for simulations on orthopedic procedures, such that the biomechanical consequences of these procedures could be studied on the computer.^2^

The interest in this subject among the medical community can be gauged from the large number of papers relating to this that are available in the literature. Today, a search in the PubMed database using the term “medical simulation training” retrieves a total of 4,360 articles. The first of these dates from the 1960s, with a notable increase in concentration over the last few years. The literature provides information on viable and efficient models and simulators destined for teaching about cardiopulmonary resuscitation, vascular accesses, video laparoscopy, anesthesia procedures and techniques within obstetrics, endoscopy and orthopedics, and so on.

In addition to the papers describing the various methods, such as the study by Savata,^3^ which described the use of virtual reality for surgical training, other studies have aimed to demonstrate what the real impact on learning would be. For example, the study by Grantcharov et al.^4^ examined the use of a virtual reality simulator to improve resident physicians’ ability to learn how to perform cholecystectomy by means of video laparoscopy, through a randomized trial on 16 students. Although their results are questionable, they indicate that the students trained with a simulator presented improvements in performance, with regard to shorter time taken to carry out the procedure (P = 0.021) and a lower rate of unnecessary movements (P = 0.003).

In the 1990s, Brazilian medical schools started to increase their interest in simulation as a teaching method. Dissemination of courses such as Advanced Cardiovascular Life Support (ACLS) and Advanced Trauma Life Support (ATLS) throughout the national territory can be noted as a major factor in spreading the use of simulated situations in this country, especially using dummies. In 2009, the School of Medicine of the University of São Paulo (Faculdade de Medicina da Universidade de São Paulo, FMUSP) inaugurated a center known as the Skills and Simulation Laboratory, which would have the purpose of providing classes on “high-fidelity simulation”. Its structure consists of six rooms that are equipped for different objectives, with 40 types of dummies and a high-technology communication system that makes it possible to record the attendance provided and study it in debriefing sessions. Several academic disciplines share this space for teaching and training in different medical techniques and competencies. Another initiative within FMUSP was implemented by the Discipline of Urology, which set up a mixed laboratory with virtual reality simulators and a structure for experimental surgery on pigs, for training on laparoscopy. The results from this regarding training improvements were published in 2011.^5^

The biggest advantages from simulation are the possibilities of providing training without involving patients and enabling repetitive training and the possibility for instructors to make better assessments of their students’ skills. Despite the high cost of simulators, they can, if correctly used, reduce the total cost of the learning process through avoidance of complications and improper use or waste of medical materials. This is especially important in the case of techniques involving expensive and fragile materials or equipment. Another advantage is that cadavers are not used and experimentation on animals is not required, which reduces the difficulties relating to acquiring such materials and the possible ethical conflicts within the training.

Even with the wide-ranging investigations on simulations that can be consulted in the literature, there are still no definitive data to prove whether any acceleration of the learning process exists, whether the safety of patients involved in the learning process is improved, or even less so, whether any gain in physicians’ final ability to practice has been achieved upon conclusion of their learning process. Nevertheless, for all the reasons that have been given, regardless of whether simulation is done on mechanical models like the dummies used for cardiopulmonary resuscitation, or on totally digital virtual reality simulators, it is a form of teaching that unequivocally adds quality to training. In the final analysis, this may produce improvements to the attendance provided for patients.

The aphorism coined by Halsted in 1904 to summarize what surgeons’ training should consist of, i.e. “see one, do one, teach one,”^6^ seems to be gradually undergoing modification to “see one, then simulate, simulate and simulate, before doing one”.
